# Medical School in Michigan

**Published:** 1846-12

**Authors:** 


### MEDICAL SCHOOL IN MICHIGAN.

We learn that efforts are now being made for the establishment of a medical school, near the central point of the State of Michigan. The persons concerned in it are gentlemen of highly respectable characters, and all that is required for perfect success of the enterprise, is prompt and vigorous action.

